# Dimensions of the foveal avascular zone using the Heidelberg retinal angiogram-2 in normal eyes

**DOI:** 10.4103/0301-4738.73706

**Published:** 2011

**Authors:** Deepa John, Thomas Kuriakose, Suresh Devasahayam, Andrew Braganza

**Affiliations:** Department of Ophthalmology, Christian Medical College, Vellore – 632002, Tamil Nadu, India; 1 Department of Bioengineering, Christian Medical College, Vellore – 632002, Tamil Nadu, India

**Keywords:** Fluorescein angiography, foveal avascular zone, Heidelberg Retinal Angiogram-2

## Abstract

**Purpose:**

The purpose was to study the dimensions of the foveal avascular zone (FAZ) using Heidelberg Retinal Angiogram-2 (HRA-2; Heidelberg Engineering GmBH, Dossenheim, Germany).

**Materials and Methods:**

An observational study of the FAZ area and circumference was done with fundus fluorescein angiography (FFA) using HRA-2 in 31 normal individuals. The FAZ was studied using both contrast-adjusted and nonadjusted methods. Contrast adjustment was done to obtain better visualization of the finer capillaries around the fovea enabling more precise measurements of the FAZ in normal eyes.

**Results:**

The mean area of the FAZ calculated by the contrast-adjusted method was 0.2753 mm^2^ (±0.074) and the mean circumference was 2.22 mm (±0.048). By the conventional method, the area and circumference of the FAZ were 0.6241 mm^2^ (±0.177) and 3.23 mm (±0.454), respectively.

**Conclusion:**

The measurements of area and circumference of FAZ using contrast-adjusted methods were significantly smaller than the conventional method.

*In vivo* and *in vitro* methods used to measure the foveal avascular zone (FAZ) show variations.[1–4] An understanding of the correct dimensions of the FAZ is important both for diagnostic and therapeutic purposes. The size of the FAZ which is supplied by the choroidal circulation determines the amount of retained vision in central retinal artery occlusions if the cilioretinal artery is spared. It also determines the safe size of the laser spot when treating near the fovea and making interpretations of angiograms.

The scanning laser of Heidelberg Retinal Angiogram-2 (HRA-2; Heidelberg Engineering GmBH, Dossenheim, Germany) captures the early phases consistently and affords a detailed study of the perifoveal capillary filling. Studies of the FAZ done by flash fundus fluorescein angiography (FFA) and analyzed by visual interpretation have limitations.

We used contrast-adjusted methods to measure the FAZ in FFA done using HRA-2. This may provide better visualization of finer capillaries around the fovea, enabling more precise measurements of the FAZ in normal eyes. This study was an attempt to establish normative data and the normal limits of variability in the size of the FAZ using HRA-2.

## Materials and Methods

Patients attending our out-patient clinic were invited to volunteer for this study. Our study was approved by our institutional review board and was done from February to June 2005 using HRA-2.

In HRA-2, the video signal generated by a scanning laser ophthalmoscope is converted into digital information. Confocality enhances image detail, contrast, and sharpness. HRA-2 has the ability to acquire dynamic, high-speed movies (up to 16 frames per second), thus facilitating documentation of the early filling stages of the retina (HRA-2).

Volunteers were explained in detail about the possible complications of FFA and written informed consent was obtained from each one of them. Baseline parameters obtained were blood pressure, pulse rate, best-corrected visual acuity, keratometry reading, axial length measurement, color vision recording, Amsler grid charting, and Humphrey Field Analyzer using program 10-2.

Inclusion criteria were subjects aged 20–40 years with best-corrected vision of 20/20 J1 in both eyes. Exclusion criteria were refractive error more than +2.00 diopter (D) or –2.00 D, presence of diabetes, hypertension and renal diseases, or intake of systemic medications like chloroquine, vasodilators, and vasoconstrictors.

**Figure 1:**
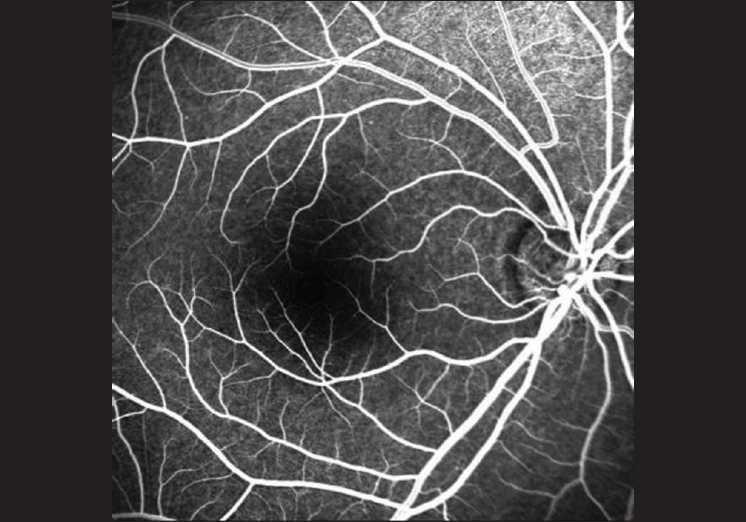
Foveal avascular zone without contrast adjustment

**Figure 2:**
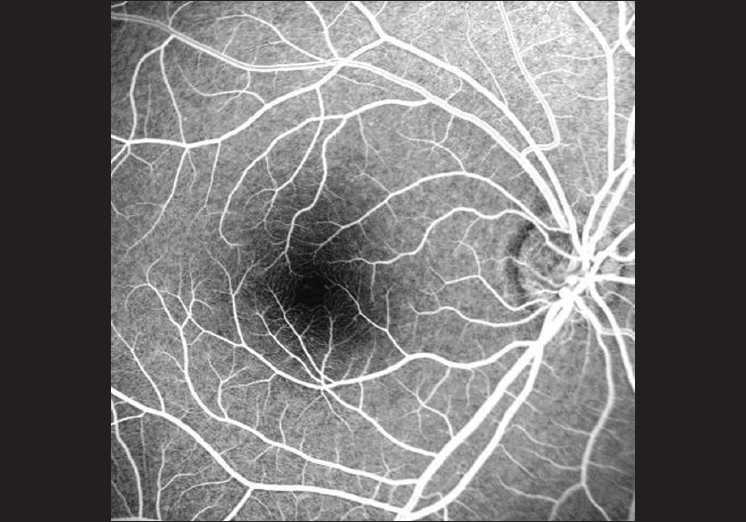
Contrast-adjusted picture of the foveal avascular zone

Three milliliters of 20% fluorescein was injected in 3 s via a scalp vein needle inserted into the dorsal vein of the hand. A seconds’ stopwatch was used to time the duration from the start of the injection of the dye. Fundus video imaging by HRA-2 was started 7 s after the injection of the dye. The video capture was done for 20 s. From this, a single image was selected for each patient at 7–9 s after the dye was seen in the first central retinal artery bifurcation to measure the FAZ [Fig. 1]. This corresponded to the arteriovenous phase. The contrast of these images was enhanced using the GNU Image Manipulation Program (GIMP). Image contrast was improved using manual histogram equalization in GIMP. The criterion for improvement was best visualization of the FAZ. This was initially done manually on four of the best images, adjusting the pixel intensity of the input and output. Pixel intensities thus obtained were mapped on a nonlinear graph (input/output intensity transformation graph). The input output values in the X and Y axes were noted and then applied to all the other images [Fig. 2]. After such contrast adjustment, the images were saved and read into an image analysis program written in our lab (using Java). This program allows the user to manually mark an area using the mouse. The area so marked would then have the enclosed area calculated in pixels by the program. Knowing the optical magnification, the area in pixels can be converted to square millimeters. We used a standard optical magnification of one pixel area, i.e., 100 × 10^-6^ mm^2^.

Statistical analysis was made using intraclass coefficient (ICC). The ICC compared the FAZ area and circumference measured by standard and the contrast-adjusted method.

## Results

The study included 31 healthy subjects, 15 males and 16 females (mean age 29.7 years).

The mean area of the FAZ calculated by the contrast-adjusted method was 0.2753 ± 0.074 mm^2^. The vertical diameter was 0.579 ± 0.015 mm and the mean circumference was 2.22 ± 0.048 mm. By the conventional method, the area and circumference of the FAZ measured were 0.6241 ± 0.177 mm^2^ and 3.23 ± 0.454 mm, respectively.

The measurements of area and circumference of the FAZ using contrast-adjusted methods were significantly smaller than the conventional method, and there was no correlation between the two methods (ICC = –0.002, *P* = 0.502).

There was no statistically significant correlation between the area of the FAZ measured and subjects’ age, blood pressure, pulse rate, and foveal threshold.

## Discussion

Diverse methods have been used to analyze the FAZ area and diameter. Variations in the measurements of the FAZ in the literature suggest that it may be affected by factors such as age, ethnicity, and eye size. Table 1 shows the comparative values of the FAZ from various studies.[1–4]

**Table 1:** Area of the foveal avascular zone (other studies)

Reference	Method	*n*	Area (mm^2^) Mean (SD)
Bresnick *et al*.[1]	FFA	20	0.35*
Wolf *et al*.[2]	FFA	21	0.231 (0.06)
Mansour *et al*.[3]	FFA	27	0.405 (0.559)
Arend *et al*.[4]	FFA	52	0.205 (0.062)

FFA: Fundus fluorescein angiography,

* Median value

To the best of our knowledge, few studies have been reported measuring the FAZ in the normal population using HRA-2. Our study was done on the Indian population. Analogous to racial variations seen in the thickness of the choroid, it is possible that there are racial variations in measurements of the FAZ. However, this has not been documented in previous reports.

The digital nature of the pictures enables us to manipulate them compared to conventional photographic FFA. The larger FAZ seen with the unadjusted picture is probably due to the loss of detail owing to the lack of contrast. It is unlikely that this reduction in area is an artifact because there is no change in the size of the picture with the adjustments. In addition, there have been other studies where the FAZ area approaches the value from this study.[2] Theoretically, trypsin digest measurements should give the most accurate value but this technique unfortunately destroys the finest of capillaries.[1] Studies on the FAZ are important to define normal values. A good description and understanding of the normal anatomy is important to interpret the FAZ in pathophysiology and plan laser treatment.

In conclusion, the contrast-adjusted method could be a tool used to study digital angiogram pictures. In our study, the FAZ area by the conventional method was 0.6241 ± 0.177 mm^2^ and by contrast-adjusted method was 0.2753 ± 0.074 mm^2^.
